# Effect of low-level laser therapy versus calcium hydroxide intra-canal medication on postoperative pain and inflammatory mediator reduction in symptomatic apical periodontitis: a randomized clinical trial

**DOI:** 10.1186/s12903-026-08642-7

**Published:** 2026-05-25

**Authors:** Radhwa Refaat Otaify, Sarah Samir Abouelenien, Angie Galal Ghoneim, Olfat Gamil Shaker

**Affiliations:** 1 Department of Endodontics, Faculty of Dentistry, Cairo University, Giza, Egypt; 2 Department of Biochemistry and Molecular Biology, Faculty of Medicine, Cairo University, Giza, Egypt

**Keywords:** LLLT; Biomodulation, Calcium Hydroxide, Post-operative Pain, IL-8, Substance P, Biomarkers

## Abstract

**Background:**

Post-operative pain following root canal therapy remains a prevalent clinical issue, especially in cases of symptomatic apical periodontitis, where inflammatory mediators such as interleukin-8 (IL-8) and substance P are critically involved in the pathophysiology. This study aimed to assess and compare the efficacy of LLLT and Calcium Hydroxide (CH) as intracanal medication in alleviating post-operative pain, percussion sensitivity, and in reducing the levels of IL-8 and substance P in periapical exudate one week after root canal instrumentation in patients with symptomatic apical periodontitis.

**Methodology:**

This comparative, parallel-design randomized clinical trial evaluated the effectiveness of low-level laser therapy (LLLT) versus Calcium Hydroxide in managing post-operative pain in 48 patients with symptomatic apical periodontitis. Patients aged 20–55 with mature, single-canalled permanent teeth were randomly assigned (1:1) to either treatment group using computer-generated randomization with concealed allocation. Root canal therapy was performed over two visits, with pre- and post-operative pain and percussion sensitivity recorded at different time intervals. Periapical fluid samples were collected immediately post-instrumentation (PS-1) and after one week (PS-2) to quantify substance P and IL-8 using ELISA. Blinding was maintained for the molecular biologist and the statistician. Data were analyzed using SPSS v25.0.

**Results:**

Two patients were lost to follow-up; therefore, 46 patients were included in the analysis. Statistical analysis revealed no significant differences between the LLLT and Calcium Hydroxide groups in terms of postoperative pain at any assessed time point, nor in percussion pain at 1-week post-instrumentation (p > 0.05). Furthermore, the reduction in substance P and IL-8 concentrations between PS-1 and PS-2 samples did not differ significantly between groups (p = 0.123 and p = 0.385, respectively).

**Conclusion:**

Within the Limitations of this study, it may be concluded that LLLT may be considered a viable single-visit treatment option and an effective alternative to Calcium Hydroxide for the management of Symptomatic Apical Periodontitis, as both demonstrated comparable clinical pain relief and biochemical modulation of IL-8 and Substance P.

**Trial registration number:**

NCT04594317 on Clinicaltrials.gov on 13th of oct. 2020.

**Supplementary Information:**

The online version contains supplementary material available at 10.1186/s12903-026-08642-7.

## Introduction

Post-operative pain remains a prevalent complication following endodontic therapy, with incidence rates reported between 3% and 58%, as highlighted by Sathorn, Parashos [1]. Multiple studies have explored various factors influencing this type of pain after root canal treatment (RCT). Among these factors, pre-operative pain, particularly in conditions such as symptomatic irreversible pulpitis and symptomatic apical periodontitis, has consistently been identified as a strong predictor of post-operative pain severity [2].

Pain and inflammation arising from apical periodontitis (AP) is primarily triggered by intra-radicular bacteria and their byproducts. Early immune responses involve periapical neutrophils releasing mediators like leukotrienes and prostaglandins to limit infection and recruit additional immune cells, including macrophages. Among these mediators is Interleukin-8 (IL-8), which is a key proinflammatory mediator, that directs granulocytes and other immune cells to the infection site, promotes osteoclast activation, and contributes to periapical bone resorption, abscess formation, and neutrophil activation through enzyme release and superoxide generation [3].

The process of apical periodontitis was further elaborated by the introduction of neurogenic inflammation concept, which was described by Bayliss in 1901 as vasodilation that occurs after noxious stimulation. This process involves the activation of nociceptors and the subsequent release of neuropeptides such as substance P (SP), which trigger vasodilation, increased vascular permeability, and mast cell degranulation [4] .The resulting histamine release amplifies nociceptive signaling, while SP stimulates macrophages to produce inflammatory mediators, including prostaglandins and pro-inflammatory cytokines (e.g., IL-1, IL-6, TNF), thereby perpetuating a cycle of inflammation and pain [5].

Various strategies have been employed to mitigate post-endodontic pain, including long-acting anesthetics, pre-emptive analgesics, and the use of intracanal medicaments, with Calcium Hydroxide being the most common. More recently, low-level laser therapy (LLLT) has emerged as a promising adjunct due to its analgesic, antimicrobial, and tissue-healing properties, coupled with a favorable safety profile. LLLT, developed by Mester in 1968, utilizes non-thermal red or near-infrared light within the 600–1000 nm range, and is thought to exert effects through modulation of ATP production and oxidative stress [6] .

This randomized clinical trial investigated the effect of low-level laser therapy (LLLT) as compared to Calcium Hydroxide (CH) intracanal medication in managing postoperative and percussion pain, as well as in modulating periapical levels of substance P and interleukin-8 (IL-8), in patients with symptomatic apical periodontitis. While previous literature has examined these treatments independently, this is the first study to undertake a direct comparison between Low-Level Laser Therapy (LLLT) and calcium hydroxide intracanal medication, providing a concomitant evaluation of clinical pain outcomes and inflammatory biomarker expression in symptomatic apical periodontitis. The null hypothesis stated that no significant differences would be observed between the two treatments in terms of pain relief or biochemical modulation.

## Materials and methods

This randomized clinical trial has been written according to CONsolidated Standards Of Reporting Trials (CONSORT) 2025 guidelines.

The study employed a comparative parallel-design randomized clinical trial design to compare two endodontic treatment interventions.

Ethical approval was obtained from the Institutional Review Board of the Faculty with Approval Number: [30-10-20], dated [27th of Dec-2020], and protocol registration was carried on *Clinicaltrials.gov* with ID [NCT04594317] on [13th of Oct. 2020].

The study’s sample size was calculated based on the primary outcome, which was the intensity of postoperative pain. Using data from Memon et al. [7]^,^ We determined that a total sample size of 48 patients (24 per group) was required to detect a significant difference between groups. This calculation was performed using PS software (version 3.1.6) with an uncorrected chi-square test, a significance level (α) of 0.05, and 80% power, while also accounting for a 15% anticipated dropout rate.

Participants were randomly assigned to either the LLLT or control group in a 1:1 ratio using a computer-generated sequence (http://www.random.org/). To ensure allocation concealment, the sequence was managed by an assistant supervisor who was the only individual aware of the assignments. This supervisor prepared identically sealed, opaque envelopes containing the participant’s number. Participants selected an envelope, and the group assignment was only revealed upon contacting the supervisor. This protocol ensured that neither the investigators nor the participants could influence the randomization process prior to treatment. While participant blinding to treatment intervention wasn’t possible, they didn’t know which intervention was considered more effective. The molecular biologist and statistician remained blinded to ensure unbiased outcome assessment and data analysis.

Eligible participants were healthy individuals aged 20–55 with mature, single-canalled permanent teeth (including both anterior and single-rooted posterior teeth) diagnosed with symptomatic apical periodontitis and percussion pain. They needed to understand the VAS, consent, and be willing to participate. Exclusion criteria included medical conditions, pregnancy, recent analgesic/antibiotic use, bruxism, and teeth with abscesses, swelling, mobility (Grade II+), deep pockets, non-restorable conditions, immature apices, or radiographic signs of resorption, fractures, perforations, or calcifications.

**Fig. 1:**
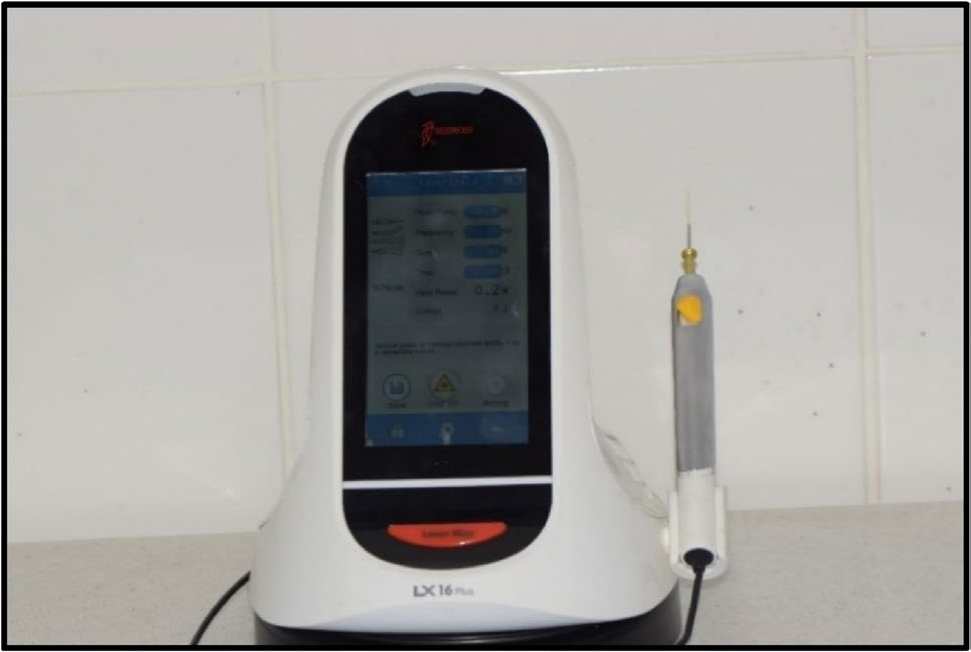
Laser LX 16 plus device with the adjusted laser parameters

**Fig. 2:**
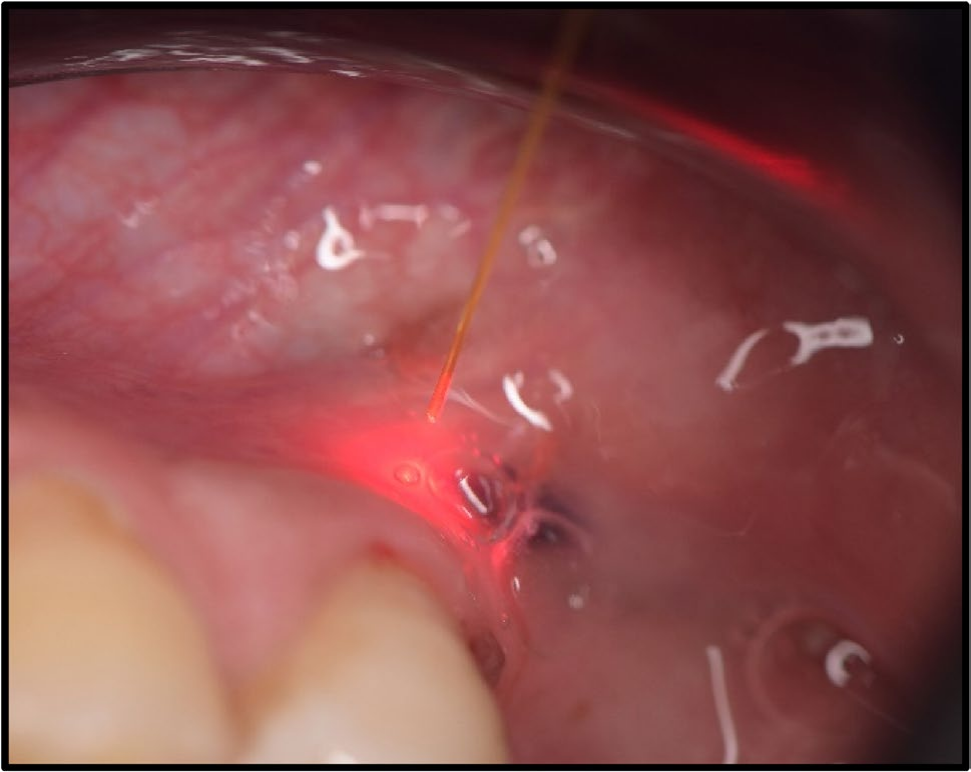
Application of optical tip at the buccal tissue around the apex of the root at a distance of approximately 10 mm with a circular movement

**Fig. 3:**
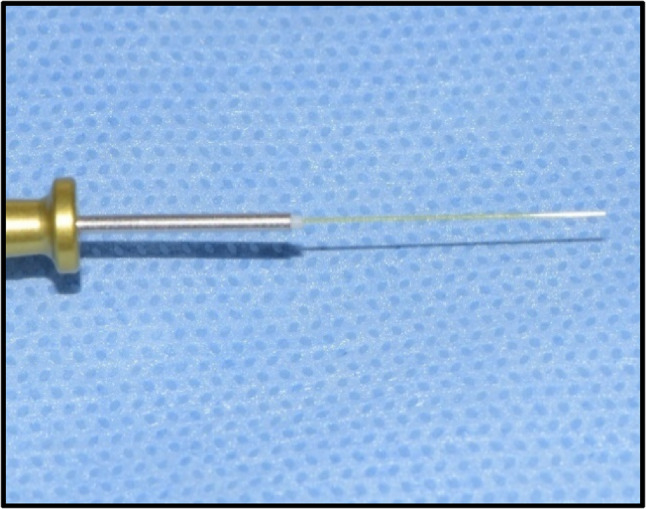
The 200-μm optical tip used for laser application

Forty-eight participants were recruited from clinic of endodontics at Faculty of Dentistry, Cairo University, Urban area, Cairo governorate, Egypt. After the final diagnosis of symptomatic apical periodontitis was reached and the patient was confirmed eligible to be enrolled in the study, the treatment was done on two visits. Assessment of preoperative pain and preoperative percussion pain levels was done at the first visit prior to treatment initiation using modified VAS. The two-visits endodontic treatment was carried out as follows:

Local anesthesia was administered via buccal infiltration using 1.8 ml of 4% Articaine with 1:100,000 epinephrine (Artinibsa^®^; Inibsa Dental, Lliçà de Vall, Spain) followed by caries and coronal restorations removal using a sterile bur. Rubber dam (Dental Dam, Sanctuary Dental, UK) isolation was then applied and the operative field (tooth, clamp, rubber dam) was disinfected using 5.25% sodium hypochlorite NaOCl (Dent House, Medical company, Cairo, Egypt). Access cavity preparation was performed using sterile carbide and Endo-Z burs (Dentsply Maillefer, Ballaigues, Switzerland). After completing the access, the operative field and the pulp chamber were cleaned and disinfected once again in the same way mentioned above. Canal patency was verified with #10 and #15 stainless steel K-files (MANI, INC., Industrial Park, Utsunomiya, Tochigi, Japan). Working length (WL) was determined using an electronic apex locator E-pex Pro (Eighteeth, Changzhou, Jiangsu, China), then confirmed radiographically to be 0.5 mm shorter than radiographic apex. Canal shaping was conducted using M-Pro rotary files (Innovative materials and Devices, Shanghai, China) and endodontic motor E-connect Pro (Eighteeth, Changzhou, Jiangsu, China) in the sequence of: 18/0.09 (orifice opener), followed by 20/0.04, 25/0.06, and 35/0.06 files to working length. A continuous rotary motion was used at 500 rpm/3 Ncm for the first file and 300 rpm/1.5 Ncm for the subsequent files. Irrigation and recapitulation were done after each instrument. The canal was thoroughly irrigated with 2.5% NaOCl root canal irrigant (5 ml for 1 min) using disposable plastic syringe with side vented needle gauge 30 Steri irrigation tips (Diadent, Chungcheongbuk-do, Korea) reaching 1 mm short of the working length. All teeth received the same volume of irrigant (5 ml prior to instrumentation, 5 ml between each file and 5 ml as final flush after root canal instrumentation to reach a total volume of 25 ml in total). The canal was then dried by using sterile paper points #35 (Meta Biomed Co. Ltd, Korea) and then flushed with 5 ml of saline to inactivate the NaOCl. The post-instrumentation periapical sample (PS-1) was collected after cleaning and shaping by introducing a fine sterile size 20 paper point (Meta Biomed Co. Ltd, Korea ) 2 mm beyond the canal terminus for 1 min [8]. This procedure was performed three times. The paper points were placed in a sterile micro-centrifugation tube (Merck, Rahway, New Jersey, US.) and immediately transferred to a 80 °C freezer (Fisher scientific, Waltham, Massachusetts, US.) until further processing. The patients were then assigned into two groups (*n* = 24), as follows:

- Intervention Group (LLLT Group): 976 ± 20 nm diode laser Laser LX16 plus (Woodpecker, Wroclaw, Poland) was activated at 0.5 W and 10 Hz Fig. (1) at approximately 10 mm to the buccal tissue around the apex of the root. This distance was standardized using a calibrated spacer Fig. (2). A circular movement was performed during application. Pulse duration was 0.5 s, and pulse pause was 50%. Total application time was 30 s for the tooth. For this application, a 200-µm optical tip was used Fig. (3). The Woodpecker LX16 device underwent factory calibration prior to the study. All LLLT applications were performed by a single operator to eliminate inter-operator variability. The LLLT protocol followed the parameters established by Arslan et al. (2018); specifically, the laser was applied for 30 s per root. While Arslan et al. utilized a total of 60 s for multi-rooted molars, the duration in the present study was set to 30 s for the single-rooted teeth included, thereby maintaining a consistent energy delivery per root [9].

- Control Group (Calcium Hydroxide Group): Calcium Hydroxide paste Metapaste (Meta Biomed Co., Ltd, Korea) was inserted into the canal using disposable plastic tip and placed at a distance 1–2 mm less than the working length.

Temporary sealing of the access cavity was completed using sterile cotton and MD-Temp (Meta Biomed Co., Ltd, Korea), and patients were recalled after one week. Pain scores were self-reported by patients at 6, 12, 24, and 48 h using the modified VAS. In the recall appointment after 1 week, percussion pain was reassessed. The canal was re-accessed, irrigated with saline prior to collection of the second periapical sample PS-2. To ensure equivalent canal conditions, Calcium Hydroxide was removed using copious irrigation with saline, combined with ultrasonic activation using ultra-X (Eighteeth, Changzhou, Jiangsu, China ) until the irrigant returned clear. The canals were inspected using a 6X Univet magnification loupes (Univet Loupes S.p.A, Rezzato, Italy) and periapical radiograph was taken to confirm the absence of Calcium Hydroxide residue. This ensured that both groups had equivalent intracanal conditions and cleanliness before obtaining second periapical sample. A final rinse with 5 ml of 2.5% NaOCl and 5 ml of 17% EDTA (Prevest Denpro Limited, Jammu, India) was done in both groups. Obturation was performed using a modified cold lateral compaction technique with Adseal epoxy resin sealer (Meta Biomed CO., LTD, Korea) and appropriate gutta-percha cones (Meta Biomed CO., LTD, Korea) (#35, 0.04 taper as master cone and # 25 auxiliary cones). The access cavity was restored with composite resin and occlusal contact was checked.

### Quantification of human substance P and IL-8

The levels of human Substance P and Interleukin-8 (IL-8) in periapical fluid samples were quantified using enzyme-linked immunosorbent assay (ELISA) kits (Bioassay Technology Laboratory, Zhejiang, China) (Cat. No. E1528Hu and E0089Hu, respectively). Post-instrumentation (PS-1) and pre-obturation (PS-2) samples were each eluted in 200 µl phosphate-buffered saline (PBS), vortexed, and centrifuged at 4000×g for 10 min. ELISA plates, pre-coated with specific antibodies for Substance P or IL-8, captured target antigens from the samples. Biotinylated secondary antibodies and Streptavidin-HRP were subsequently added. After washing, substrate solution was introduced, producing a color change proportional to antigen concentration. The reaction was stopped using an acidic solution, and absorbance was read at 450 nm. Quantification was achieved by constructing a standard curve from known concentrations, plotted against optical density values using regression analysis software, enabling precise determination of Substance P and IL-8 concentrations in the experimental samples.

### Substance P assay principle

#### Reagent preparation

**Fig. 4:**
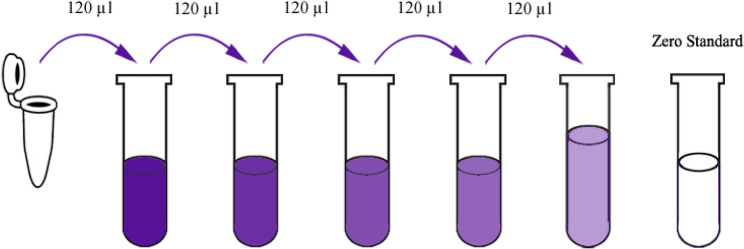
Diagrammatic representation of standard serial dilution

All reagents were equilibrated to room temperature prior to use. A standard stock solution of 1200 ng/L was prepared by reconstituting 120 µl of the 2400 ng/L standard with an equal volume of diluent. Following 15 min of gentle agitation, a serial 1:2 dilution series was performed to generate standard points at 600, 300, 150, and 75 ng/L, with standard diluent serving as the zero control (0 ng/L). The 25x wash buffer concentrate was diluted with distilled water to a 1x working concentration. Tables (1 and 2) and Fig. 4.

**Table 1:** Serial dilution of the standard (substance P ELISA kit)

1200ng/L	Standard No.5	120 µl Original Standard + 120 µl Standard Diluent
600ng/L	Standard No.4	120 µl Standard No.5 + 120 µl Standard Diluent
300ng/L	Standard No.3	120 µl Standard No.4 + 120 µl Standard Diluent
150ng/L	Standard No.2	120 µl Standard No.3 + 120 µl Standard Diluent
75ng/L	Standard No.1	120 µl Standard No.2 + 120 µl Standard Diluent

**Table 2:** Concentration of different standard solutions (substance P ELISA kit)

Standard Concentration	Standard No.5	Standard No.4	Standard No.3	Standard No.2	Standard No.1
2400ng/L	1200ng/L	600ng/L	300ng/L	150ng/L	75ng/L

#### Assay procedure and incubation

The assay was performed using a pre-coated Human SP antibody plate. To the standard wells, 50 µl of the prepared standards (containing biotinylated antibody) were added. Sample wells received 40 µl of sample followed by 10 µl of anti-SP antibody. Subsequently, 50 µl of streptavidin-HRP was added to both sample and standard wells. After thorough mixing, the plate was sealed and incubated for 60 min at 37 °C. The plate was then washed five times with 0.35 ml of wash buffer per well, with 30–60 s soak times, and blotted dry.

#### Color development and detection

Following the wash cycle, 50 µl each of substrate solutions A and B were added to every well. The plate was sealed and incubated in the dark for 10 min at 37 °C to allow for color development. The reaction was terminated by adding 50 µl of stop solution, resulting in an immediate color change from blue to yellow. The optical density was measured via a microplate reader at 450 nm within 10 min of adding the stop solution.

### IL-8 assay principle

#### Reagent preparation

**Fig. 5:**
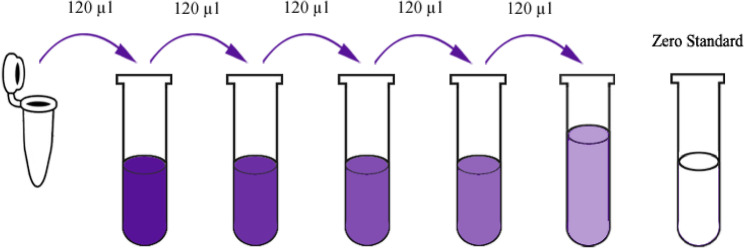
Diagrammatic representation of standard serial dilution

All reagents were equilibrated to room temperature prior to use. A 640 ng/L standard stock solution was prepared by reconstituting 120 µl of the 1280 ng/L standard with an equal volume of diluent. After 15 min of gentle agitation, a 1:2 serial dilution series was performed in duplicate to generate standard concentrations of 320, 160, 80, and 40 ng/L, with standard diluent serving as the zero control (0 ng/L). The 25x wash buffer concentrate was diluted with distilled water to a 1x working solution. Tables (3 and 4) and Fig. 5.

**Table 3:** Serial dilution of the standard (IL-8 ELISA kit)

640ng/L	Standard No.5	120 µl Original Standard + 120 µl Standard Diluent
320ng/L	Standard No.4	120 µl Standard No.5 + 120 µl Standard Diluent
160ng/L	Standard No.3	120 µl Standard No.4 + 120 µl Standard Diluent
80ng/L	Standard No.2	120 µl Standard No.3 + 120 µl Standard Diluent
40ng/L	Standard No.1	120 µl Standard No.2 + 120 µl Standard Diluent

**Table 4:** Concentration of different standard solutions (IL-8 ELISA kit)

Standard Concentration	Standard No.5	Standard No.4	Standard No.3	Standard No.2	Standard No.1
1280ng/L	640ng/L	320ng/L	160ng/L	80ng/L	40ng/L

#### Assay procedure and incubation

The assay utilized a plate pre-coated with Human IL-8 antibody. 50 µl of each standard was added to designated wells. For samples, 40 µl of sample was added, followed by 10 µl of anti-IL-8 antibody. Subsequently, 50 µl of streptavidin-HRP was added to all standard and sample wells. The plate was sealed, mixed thoroughly, and incubated for 60 min at 37 °C. Following incubation, the plate was washed five times with wash buffer and blotted against absorbent material.

#### Color development and detection

To each well, 50 µl of substrate solution A and 50 µl of substrate solution B were added. The plate was covered and incubated in the dark for 10 min at 37 °C to allow for color development proportional to the IL-8 concentration. The reaction was terminated by adding 50 µl of acidic stop solution, shifting the color from blue to yellow. The optical density was measured at 450 nm using a microplate reader within 10 min of adding the stop solution.

### Protocol deviations

While the study largely adhered to the registered protocol, minor deviations occurred regarding the laser model, age range, paper-point sampling technique and reported outcomes.

**Table:** 

Parameter	Protocol (NCT04594317)	Actual Implementation	Rationale
Laser Device	Biolase Epic X	Woodpecker LX16	Institutional availability; equivalent specs.
Age Range	25–50 years	20–55 years	Facilitated representative recruitment.
Sampling	2 paper points / Saline	3 paper points / Dry	Optimized biochemical yield/stability.
Outcomes	Pain/Biomarkers	Added Correlation analyses	Provided deeper clinical-biological insight.

### Study outcome measures

#### Prespecified outcomes

- Primary Outcomes: Post-operative pain intensity was evaluated via a modified (VAS) at four specific intervals (6, 12, 24, and 48 h) and for percussion pain at 1-week post-instrumentation.
- Secondary Outcomes: Changes in periapical inflammatory markers (Substance P and IL-8) were quantified using ELISA testing, comparing levels from immediately post-instrumentation to pre-obturation (one-week post-instrumentation).

#### Exploratory analyses: supplementary correlation analyses

Correlation between IL-8 and substance P concentrations and pain intensity at both pre-operative and one-week post-operative intervals.

### Statistical analysis

Statistical analysis was conducted using SPSS software (Armonk, NY: IBM Corp) (IBM Corp. Released 2017. IBM SPSS Statistics for Windows, Version 25.0.). Normality was assessed via Shapiro-Wilk test. Non-parametric tests (Mann-Whitney U, Wilcoxon, Friedman with Dunn’s post hoc) were used for continuous data, while Chi-square tested categorical data. Significance was set at *p* ≤ 0.05. Data were reported as means, medians, and frequencies.

In addition to the primary and secondary outcomes analyses, supplementary correlation analyses were performed post-hoc to explore the relationships between clinical symptoms and biochemical markers. These correlation findings are presented as exploratory analyses to distinguish them from the a priori study objectives.

## Results

**Fig. 6:**
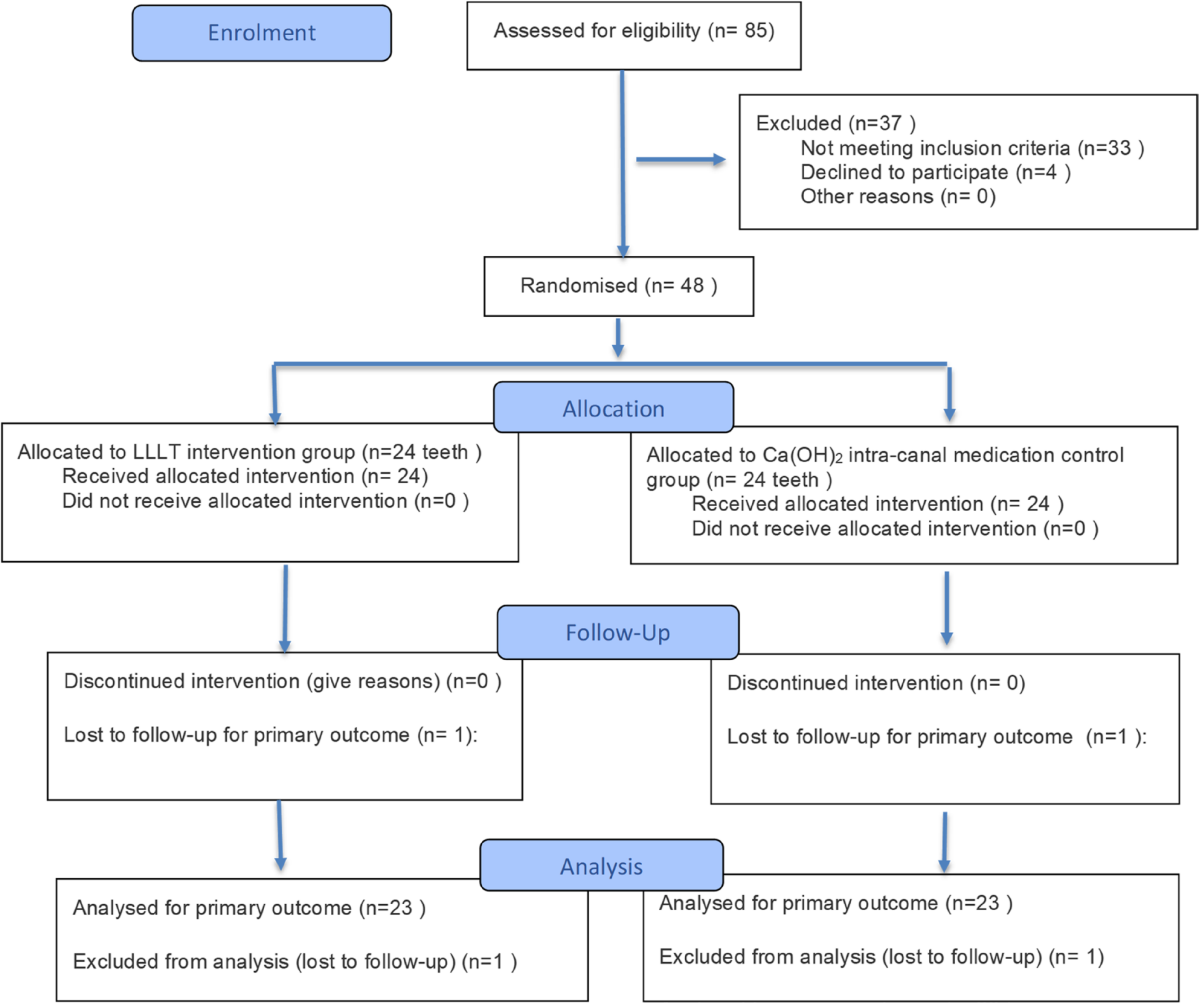
CONSORT 2025 Flow diagram of the trial design [10]

For this study, 85 patients were assessed for eligibility between December 2021 and January 2023. A total of 48 patients met the inclusion criteria and were enrolled in the study. After patients’ recruitment, randomization was done into two groups of 24 patients each. Two patients were lost to follow-up; therefore, 46 patients were included in the analysis. Flow of the patients is summarized in the CONSORT 2025 Flow diagram of the trial design presented in Fig. 6.

### Demographic data

**Fig. 7:**
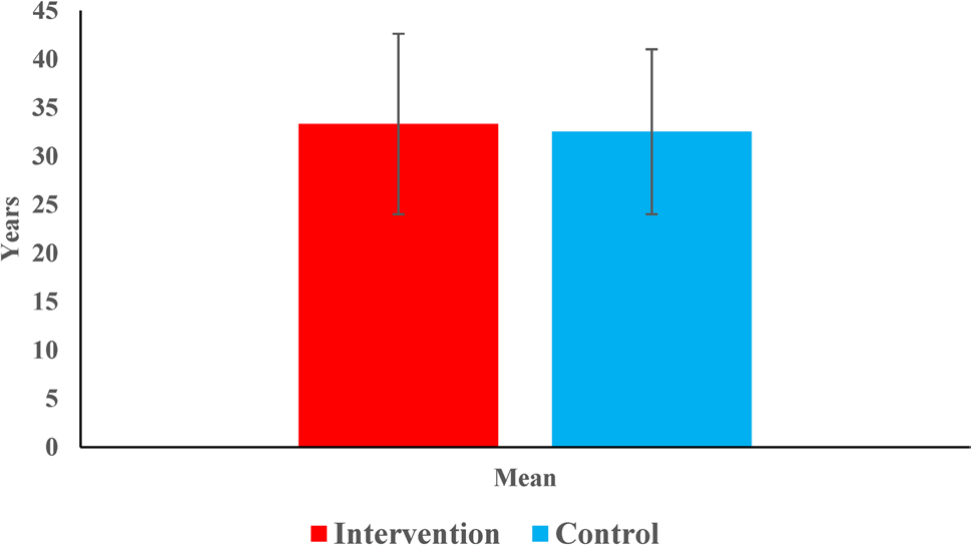
Bar chart representing mean age in both groups

**Fig. 8:**
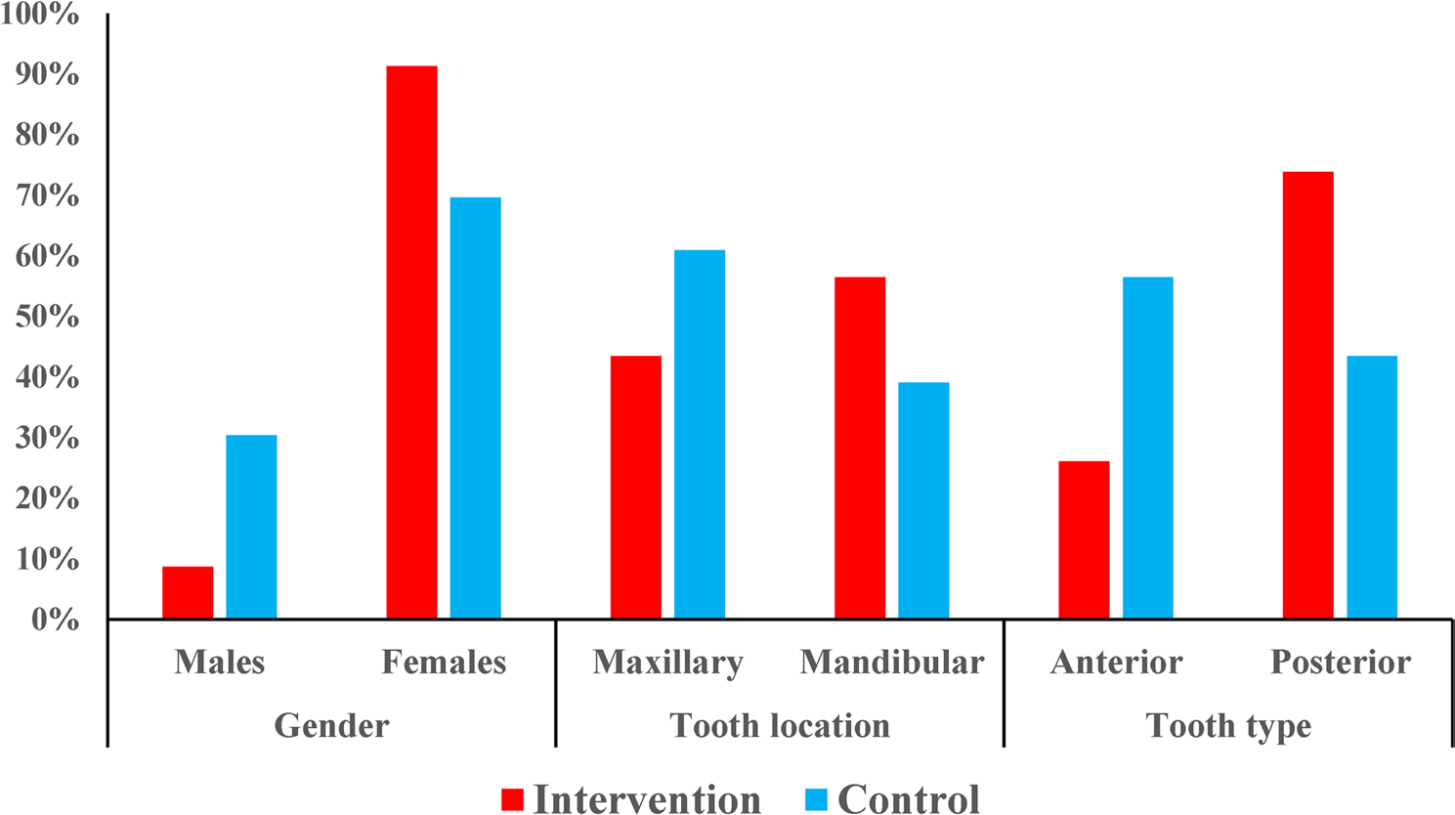
Bar chart representing gender, tooth location and tooth type distribution in both groups

The intervention and control groups were comparable in terms of age, gender, and tooth location, with no statistically significant differences observed (*p* > 0.05). The mean age was 33.3 ± 9.3 years in the intervention group and 32.5 ± 8.5 years in the control group. Females predominated in both groups, particularly in the intervention group (91.3%). Root canal treatments were more frequent in maxillary teeth in the control group and in mandibular teeth in the intervention group, though this difference was not significant (*p* = 0.194). However, posterior teeth were significantly more prevalent in the intervention group (*p* = 0.049) (Table 5) (Figs. 7 and 8).

**Table 5:** Demographic and clinical characteristics of intervention and control groups

Variable	Intervention Group (*n* = 23)	Control Group (*n* = 23)	*p*-value
Age (years)			0.603
Mean (SD)	33.3 (9.3)	32.5 (8.5)	
Median (Range)	35 (20–52)	30 (21–55)	
Gender			0.111
Males	2 (8.7%)	7 (30.4%)	
Females	21 (91.3%)	16 (69.6%)	
Tooth location distribution			0.194
Maxillary Teeth	10 (43.5%)	14 (60.9%)	
Mandibular Teeth	13 (56.5%)	9 (39.1%)	
Tooth type distribution			0.049*
Anterior Teeth	6 (26.1%)	13 (56.5%)	
Posterior Teeth	17 (73.9%)	10 (43.5%)	

*Significant at *p*=0.05

### Primary outcome data

#### Pain intensity

**Fig. 9:**
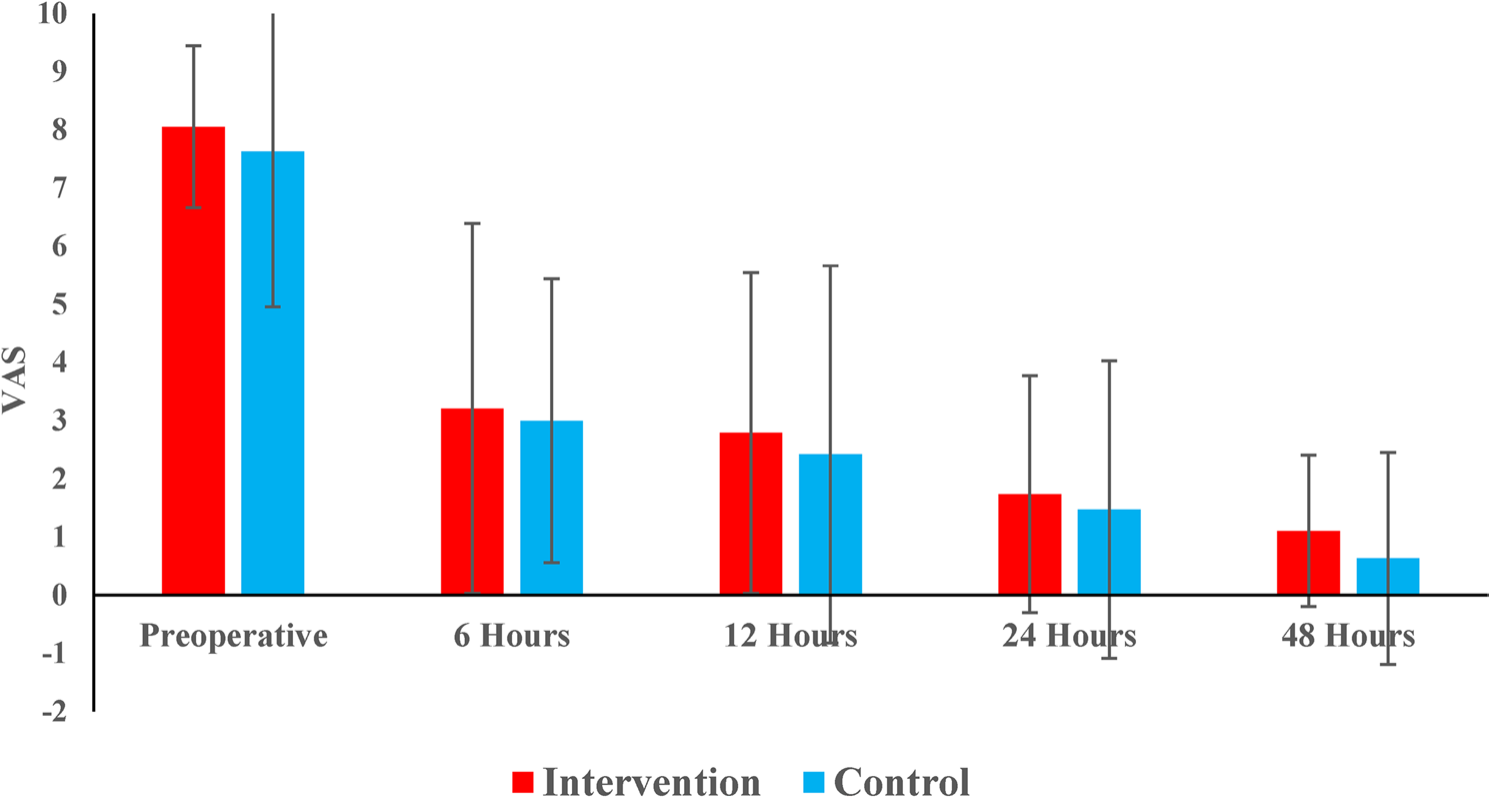
Bar chart representing the mean pain intensity at different time intervals

Pre-operative and post-operative pain intensities were evaluated in both groups. The intervention group reported a mean pre-operative pain score of 8.1 (± 1.4), while the control group had a mean of 7.6 (± 2.7), with no significant difference (p=0.840). Pain levels progressively decreased over time in both groups. At 6, 12, 24, and 48 h post-operatively, no statistically significant differences were observed between groups (p > 0.05). Both groups showed a significant reduction in pain from preoperative levels at all postoperative time points. No significant differences were found between pain levels at 6, 12, and 24 h. However, pain at 48 h was significantly lower than at 6 and 12 h in both groups (Table 6) (Fig. 9).

**Table 6:** Descriptive statistics and the results of Mann-Whitney U test with Hodges-Lehman median difference and 95% CI for comparison of pre-operative and post-operative pain intensity at different time intervals between the two groups

		Intervention	Control	*HL median difference (95% CI)*	*p*-value
Preoperatively	Mean (SD)	8.1 (1.4)	7.6 (2.7)	0 (-1, 1)	0.840
	Median (Range)	8 (5, 10)	8 (2, 10)		
6 h	Mean (SD)	3.0 (3.2)	3.2 (2.4)	0 (-2, 2)	0.773
	Median (Range)	2 (0, 8)	5 (0, 7)		
12 h	Mean (SD)	2.4 (2.8)	2.8 (3.2)	0 (-2, 1)	0.751
	Median (Range)	1 (0, 7)	2 (0, 9)		
24 h	Mean (SD)	1.5 (2.0)	1.7 (2.6)	0 (-1, 1)	0.885
	Median (Range)	0 (0, 7)	0 (0, 10)		
48 h	Mean (SD)	0.6 (1.3)	1.1 (1.8)	0 (0, 0)	0.644
	Median (Range)	0 (0, 5)	0 (0, 5)		

#### Percussion pain intensity

**Fig. 10:**
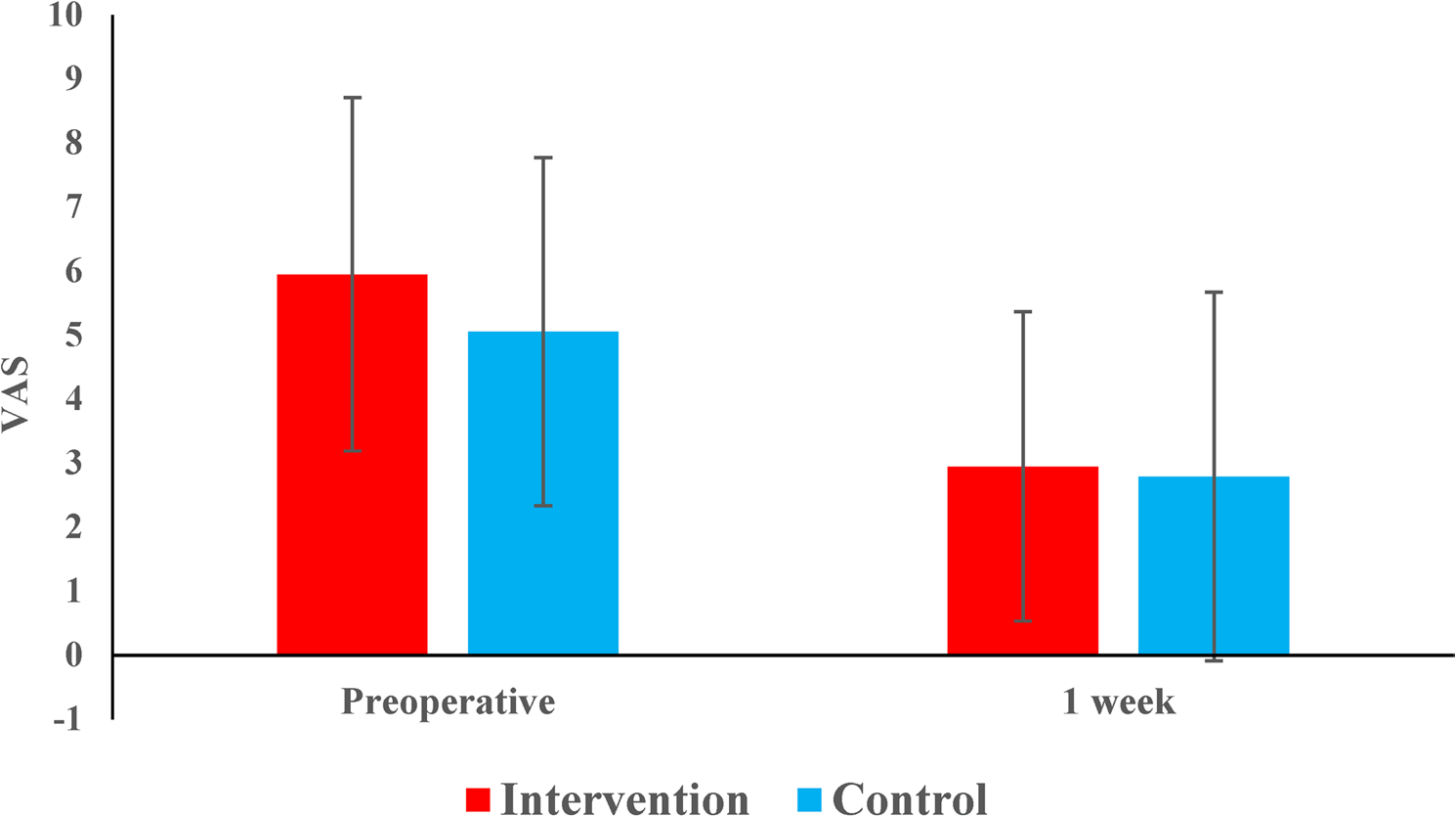
Bar chart representing the mean percussion pain intensity preoperatively and 1 week post-operatively in the two groups

Pre-operative and one-week post-operative percussion pain intensity showed no statistically significant differences between the intervention and control groups (*p* = 0.297 and *p* = 0.708, respectively). Both groups exhibited comparable mean and median values, indicating similar pain responses at both assessment time points. In both groups, a significant reduction in percussion pain intensity was observed from preoperative to one-week postoperative assessments. The intervention group showed a decrease from 5.9 ± 2.8 to 2.9 ± 2.4 (*p* < 0.001), while the control group decreased from 5.1 ± 2.7 to 2.8 ± 2.9 (*p* = 0.039) (Table 7) (Fig. 10).

**Table 7:** Descriptive statistics and the result of Mann-Whitney U test with Hodges-Lehman median difference and 95% CI for comparison of percussion pain intensity preoperatively and 1 week post-operatively between the two groups

	Descriptives	Intervention	Control	*HL median difference (95% CI)*	*p-*value
Preoperatively	*Mean (SD)*	5.9 (2.8)	5.1 (2.7)	1 (-1, 3)	0.297
	*Median (Range)*	7 (1, 10)	6 (1, 9)		
1 week postoperatively	*Mean (SD)*	2.9 (2.4)	2.8 (2.9)	0 (-1, 2)	0.708
	*Median (Range)*	3 (0, 8)	2 (0, 10)		

### Secondary outcome data

#### Reduction of periapical IL-8 concentrations (intergroup comparison)

**Fig. 11:**
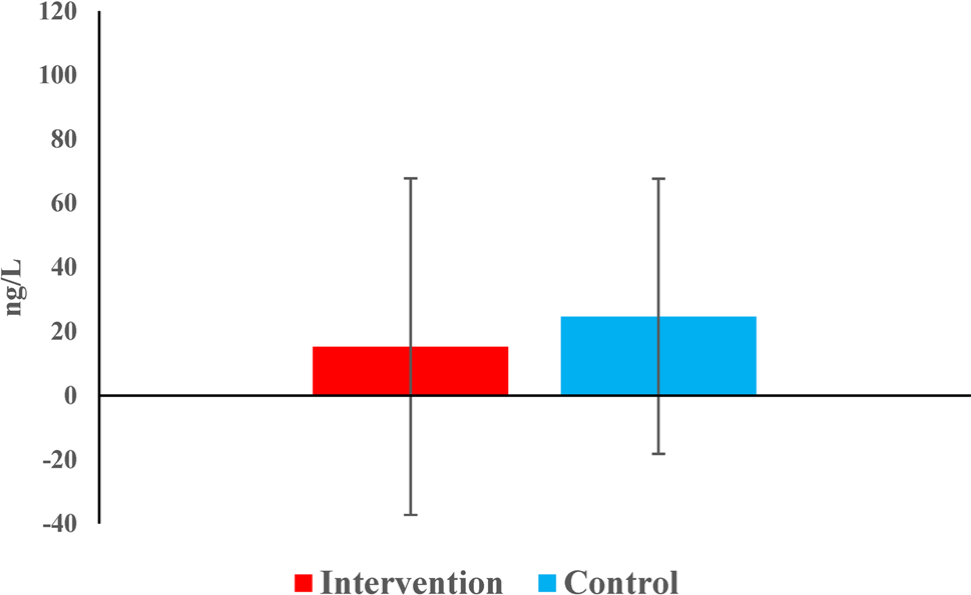
Bar chart representing the mean reduction in IL8 concentrations in the two groups

The reduction in IL-8 concentrations did not differ significantly between groups (*p* = 0.123) (Table 8) (Fig. 11).

**Table 8:** Descriptive statistics and the result of Mann-Whitney U test with Hodges-Lehman median difference and 95% CI for comparison of reduction in IL8 level between the two groups

Descriptives	Intervention	Control	HL median difference (95% CI)	*p*-value
*Mean (SD)*	15.3 (52.5)	24.7 (42.9)	-17 (-44.4, 9.7)	0.123
*Median (Range)*	3.1 (-44.7, 217)	18.3 (-72.4, 90.8)		

#### Changes in IL8 within each group (intragroup comparison)

**Fig. 12:**
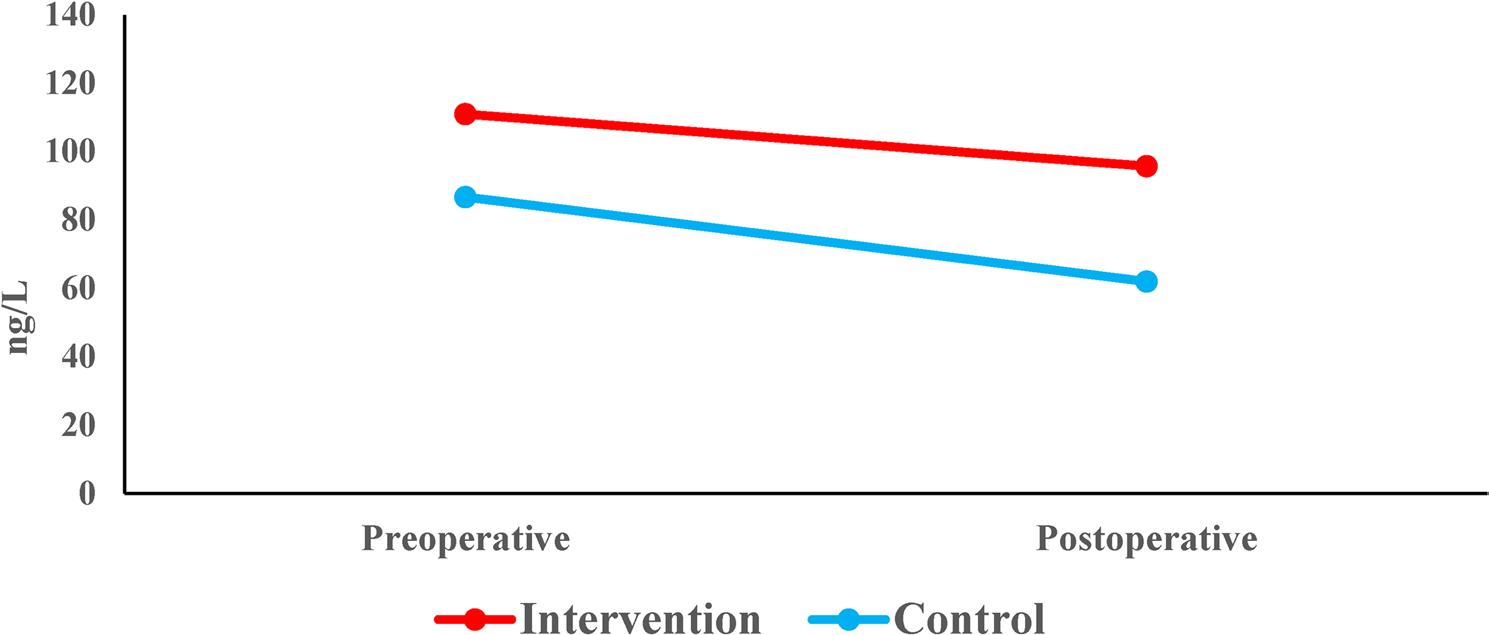
Line chart representing the change in IL8 concentration within each group

Intragroup analysis of IL-8 levels revealed divergent patterns between the two study groups. In the intervention (LLLT) group, the reduction from a baseline of 111 ± 61.6 ng/L (PS-1) to 95.7 ± 25 ng/L(PS-2) did not reach statistical significance (*p* = 0.064).In contrast, the control group demonstrated a statistically significant decrease in IL-8 concentrations, falling from 86.7 ± 20.5 ng/L (PS-1) to 61.9 ± 33.5 ng/L (PS-2) over the same period (*p* = 0.044) (Table 9) (Fig. 12).

**Table 9:** Mean (SD) and the results of Wilcoxon signed rank test with Hodges-Lehman median difference and 95% CI for comparison of the IL8 concentration before and after root canal treatment within each group

		Preoperative (PS-1)	Post-operative (PS-2)	*HL median difference (95% CI)*	*p-*value
Intervention	Mean (SD)	111.0 (61.6)	95.7 (25.0)	-6.9 (-16.1, 0.2)	0.064
	Median (Range)	100.2 (61.2, 354.1)	98.5 (31.1, 147.4)		
Control	Mean (SD)	86.7 (20.5)	61.9 (33.5)	-25.5 (-45.1, -3.3)	0.044*
	Median (Range)	87.6 (48.6, 113)	42.7 (22.2, 121.3)		

*Significant at* p*=0.05

### Reduction of periapical substance-P concentrations (intergroup comparison)

**Fig. 13:**
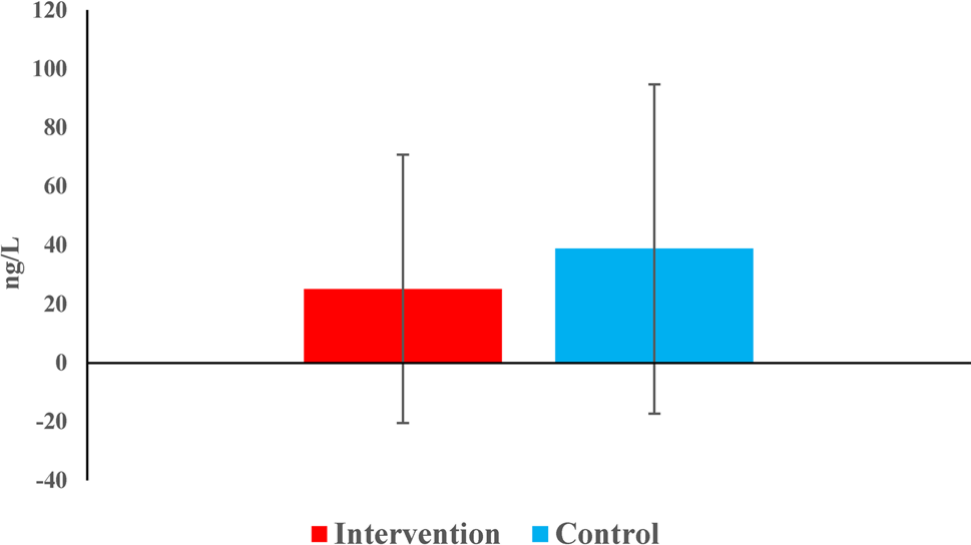
Bar chart representing the mean reduction in substance-P level in the two groups

The mean reduction was greater in the control group (38.7 ± 56.0 ng/L) than in the intervention group (25.2 ± 45.6 ng/L), however, there was no statistically significant difference between them (*p* = 0.385) (Table 10) (Fig. 13).

**Table 10:** Descriptive statistics and the result of Mann-Whitney U test with Hodges-Lehman median difference and 95% CI for comparison of reduction in substance-P level between the two groups

Descriptives	Intervention	Control	HL median difference (95% CI)	*p*-value
*Mean (SD)*	25.2 (45.6)	38.7 (56)	-14.9 (-50.7, 14.5)	0.385
*Median (Range)*	33.3 (-77.1, 123.9)	41.1 (-73.5, 115)		

### Changes in substance P within each group (intragroup comparison)

**Fig. 14:**
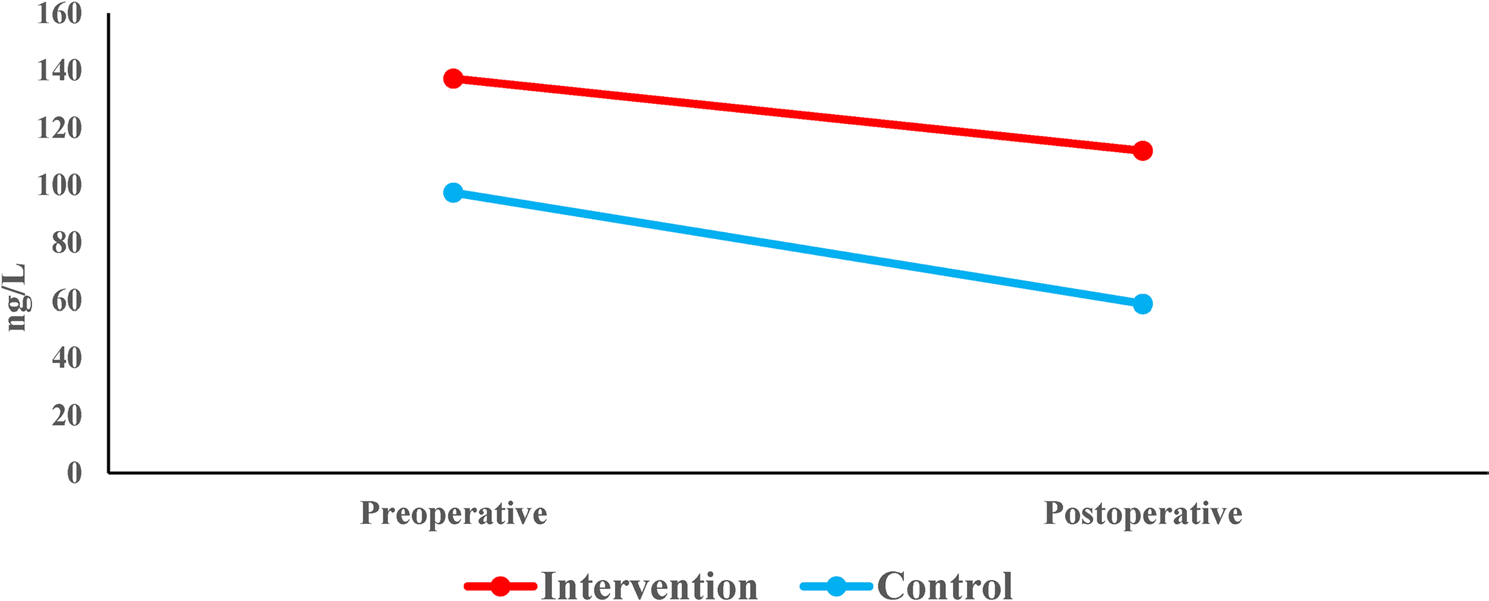
Line chart representing the change in substance P concentration within each group

Intragroup analysis demonstrated a statistically significant decline in Substance P concentrations for both study groups. In the intervention (LLLT) group, levels decreased from a baseline of 137.3 ± 34 ng/L (PS-1) to 112.1 ± 39.8 ng/L (PS-2) (*p* = 0.04). Similarly, the control group exhibited a significant reduction from 97.5 ± 30.1 ng/L (PS-1) to 58.8 ± 42.0 (PS-2) (*p* = 0.013) (Table 11) (Fig. 14).

**Table 11:** Mean (SD) and the results of Wilcoxon signed rank test with Hodges-Lehman median difference and 95% CI for comparison of the substance P concentration before and after root canal treatment within each group

		Preoperative (PS-1)	Post-operative (PS-2)	*HL median difference (95% CI)*	*p-*value
Intervention	Mean (SD)	137.3 (34.0)	112.1 (39.8)	-31.1 (-43.1, -2.7)	0.04*
	Median (Range)	141.4 (64.7, 193.5)	112 (34.4, 210.6)		
Control	Mean (SD)	97.5 (30.1)	58.8 (42.0)	-40.3 (-70.5, -10.4)	0.013*
	Median (Range)	101.4 (49.2, 146.5)	47.2 (8.5, 137.3)		

*Significant at *p* = 0.05

### Exploratory analyses: correlation of outcomes

Spearman rank-order correlation analyses were conducted to evaluate associations between concentrations of IL-8 and substance P and pain intensity at different time points. No significant correlations were observed between preoperative levels of IL-8 or substance P and either general preoperative pain intensity (rs = -0.008, *p* = 0.955; rs = 0.102, *p* = 0.541, respectively) or pain on percussion (rs = -0.014, *p* = 0.931; rs = 0.054, *p* = 0.748, respectively). Similarly, postoperative concentrations of these biomarkers showed no significant correlation with pain on percussion one week after treatment, with IL-8 (rs = 0.047, *p* = 0.779) and substance P (rs = 0.118, *p* = 0.481) both demonstrating weak or negligible associations.

## Discussion

Low-level laser therapy (LLLT), or laser bio-stimulation, is a widely used medical laser application gaining expert attention. Using low-output lasers with wavelengths between 630 and 1300 nm, LLLT penetrates deep into tissues to stimulate cell components like mitochondria. Unlike thermal therapies, it works through photon absorption, triggering cellular stimulation without generating heat—an effect known as photo-bio-stimulation^(11)^.

At the cellular level, the efficacy of Low-Level Laser Therapy (LLLT) is governed by primary and delayed photochemical reactions centered on mitochondrial activity. LLLT modulates the cellular redox state, shifting metabolism from an anaerobic to an aerobic state, which reduces the accumulation of acidic waste products that typically exacerbate pain and inflammation. This process is driven by the activation of the mitochondrial respiratory chain, leading to a surge in adenosine triphosphate (ATP) synthesis. This increase in available energy serves as a catalyst for downstream “delayed” reactions, including mRNA activation and DNA replication. The resulting protein synthesis; producing essential enzymes and cofactors, regulates cellular metabolism and fosters a biological environment conducive to healing, particularly in cells with a compromised initial redox state [11].

These cellular changes translate into broader tissue-level and neurogenic responses, categorized into primary and secondary effects. The immediate primary response involves localized vasodilation, improved lymphatic drainage, and an elevation of the pain receptor stimulus threshold, which directly mitigates acute discomfort. This is followed by secondary systemic effects where LLLT influences inflammatory mediators and neurogenic pathways. Specifically, it stimulates the production of anti-inflammatory prostaglandins (such as PGL2) and modulates the immune response through increased immunoglobulin and lymphokine activity. Crucially, the analgesic effect is further reinforced by the enhanced release of beta-endorphins and enkephalins, providing a neurogenic basis for the reduction in postoperative pain [12, 13].

The physiological benefits of LLLT appear to offer a more targeted clinical outcome than systemic pharmacotherapy. This is supported by Toopalle et al., and Kadam et al., recent systematic reviews indicating that photo-biomodulation provides superior pain modulation compared to traditional analgesics, particularly during the critical 24- to 72-hour postoperative window. By directly intervening in the inflammatory cascade, LLLT achieves a statistically significant reduction in discomfort that surpasses the efficacy of routine drug protocols. However, the consistent success of this mechanism remains dependent on the standardization of laser parameters across diverse endodontic procedures [14, 15].

Although previous studies have explored various LLLT irradiation protocols and their effects on postoperative pain, there is a paucity of evidence comparing its efficacy directly to Calcium Hydroxide intracanal medication in cases of symptomatic apical periodontitis. This research addressed this gap through a novel comparative evaluation, distinguished by the simultaneous quantification of patient-reported pain and objective biochemical markers of inflammation.

To ensure standardized and accurate sampling of periapical inflammatory mediators, only single-canalled teeth (including both anterior and single-rooted posterior teeth) were included. This anatomical simplicity facilitates direct access to the periapical tissues, whereas multi-rooted teeth were excluded to eliminate the risk of varying pathological conditions across multiple roots, which could interfere with the biochemical analysis. Teeth from both arches were included for broader applicability. Patients with preoperative and percussion pain were selected due to their link with post-endodontic pain [16]. The inclusion of both genders and relatively wide age range (20–55) were based on evidence showing them to have no significant effect on postoperative pain [17]. These criteria collectively aimed to standardize the sample while maintaining external validity.

Post-operative pain was the primary outcome due to its clinical significance and impact on patient experience. It was measured using a modified VAS at 6, 12, 24, and 48 h, aligning with typical inflammation and pain resolution patterns [18]. Pain assessment began at 6 h to exclude anesthesia effects. Secondary outcomes included percussion pain, indicating apical inflammation, and periapical levels of IL-8 and substance P, which served as objective biomarkers of inflammatory activity [19]. These combined measures provided both clinical and biological insights into treatment effectiveness.

The study’s internal validity was ensured through proper randomization, allocation concealment, and blinding of the molecular biologist and statistician, with patients unaware of treatment efficacy. Baseline comparability between groups confirmed successful randomization and reduced confounding. A validated modified VAS provided reliable pain assessment, while biomarker analysis offered objective inflammatory data. Standardized laser parameters ensured consistent intervention delivery. Appropriate statistical methods and pre-study power analysis with sample size calculation further supported data integrity and analytical rigor.

The results showed no significant differences between groups in age, gender, tooth location, preoperative pain, percussion pain, or IL-8 levels, confirming effective randomization and reducing confounding. Tooth type showed a marginally significant difference (*P* = 0.049), however, prior studies found it unrelated to postoperative pain, with only preoperative symptoms being predictive [2]. A significant difference in preoperative substance P levels (*P* < 0.001) was noted, representing a potential confounding factor that could affect interpretation of the intervention’s impact.

Post-instrumentation pain was consistently lower in the LLLT group than in the Calcium Hydroxide group at all measured intervals (6, 12, 24, and 48 h), though differences were not statistically significant. LLLT’s pain-reducing effects are linked to neural inhibition mechanisms: altering nerve conduction and nociceptor activity, and biological mechanisms, such as reducing inflammatory mediators and enhancing metabolism and drainage [20, 21]. Both groups showed significant pain reduction from baseline. These findings align with several systematic reviews and trials supporting LLLT’s analgesic benefits [22–24], though some studies reported no effect, likely due to differences in laser protocols and patient criteria [25]. Importantly, this study is unique in directly comparing LLLT to Calcium Hydroxide intracanal medication.

At the 1-week follow-up, both LLLT and Calcium Hydroxide groups showed significant reductions in percussion pain compared to preoperative levels, with the Calcium Hydroxide group showing slightly lower mean scores, though not statistically significant. Percussion pain reduction is linked to decreased periodontal ligament inflammation, which correlates more with periapical neuropeptides than spontaneous pain [26]. LLLT may relieve pain by modulating peripheral C-fibers and altering pain mediators through photochemical effects [27]. Its anti-inflammatory action, edema reduction, and improved lymphatic drainage help lower intra-periapical pressure, contributing to pain relief [28]. While some studies support LLLT’s effect on reducing percussion pain versus mock laser therapy [29, 30], others found no significant difference [28, 30]. Notably, no prior study has directly compared LLLT with Calcium Hydroxide in this context, highlighting the novelty of this investigation.

Postoperative analysis of IL-8 and substance P (SP) levels in periapical fluids showed that within LLLT no significant reduction in postoperative IL-8 was observed as compared the preoperative level, meanwhile both LLLT and Calcium Hydroxide groups had a significant reduction in post-operative SP as compared to the preoperative levels. No significant difference was observed between the two groups in the reduction of either biomarker. The lack of IL-8 reduction in the LLLT group may relate to IL-8’s dual role in healing—promoting angiogenesis and neutrophil recruitment in the early phase, but potentially delaying healing if persistently elevated [31]. The observed SP reduction with LLLT may be explained by its influence on the TRPV1 channel, which modulates pain and inflammation through sensory nerve fibers [32]. These findings contrast with previous studies reporting significant IL-8 and SP reductions with LLLT, indicating positive biomodulation [33, 34]. However, some conflicting reports noted increased SP levels post-LLLT, suggesting variability due to treatment protocols or patient conditions [35].

The lack of significant correlation between pain intensity and IL-8 or substance P levels reflects the complex, multifactorial nature of periapical inflammation. These findings must be contextualized as a function of individual variability and the inherent limitations of sampling time points. Discrepancies may arise from fluctuations in lesion severity, immune profiles, and tissue sensitivity, alongside subjective factors such as age, anxiety, and baseline pain thresholds. Furthermore, because inflammatory mediators follow specific kinetic patterns, the limited sampling windows in this study may not have coincided with the peak biochemical activity of IL-8 or substance P. This temporal mismatch, combined with the subjective nature of pain reporting, likely constrained the detection of meaningful associations between molecular markers and perceived clinical symptoms.

The findings of this study suggest that LLLT provides significant practical advantages in the management of symptomatic apical periodontitis. Specifically, the application of LLLT may enable a shift toward predictable single-visit endodontics by providing immediate analgesic and anti-inflammatory effects without the mandatory use of Calcium Hydroxide as an intracanal medicament. By eliminating the need for a second appointment, LLLT helps avoid the inherent risks associated with multi-visit therapy, such as inter-appointment flare-ups, coronal leakage of temporary restorations, and the potential for canal contamination during repeated access. Ultimately, this approach enhances patient comfort and clinical efficiency, offering a streamlined alternative to traditional postoperative pain management protocols.

This randomized clinical trial has several strengths, including its novelty in comparing LLLT with Calcium Hydroxide in managing postoperative pain, percussion pain, and inflammatory biomarkers in symptomatic apical periodontitis. Rigorous methodology included proper randomization, allocation concealment, blinding, and standardized protocols. The use of validated assessment tools and inclusion of both clinical and biochemical outcomes enhance the study’s reliability, clinical applicability, and contribution to endodontic pain management research.

Despite its strengths, this study has several limitations, including the inability to blind participants, potentially introducing performance bias. Restriction to single-canalled teeth limits generalizability, and pain assessment remains subjective despite validated tools. The sample size, while adequate, may not detect subtle biomarker associations. As the sample size was calculated based on the primary outcome (postoperative pain), it may have been underpowered to achieve a normal distribution for secondary biochemical markers. Consequently, these findings should be interpreted with caution. Future research should utilize larger cohorts specifically powered for biomarker analysis to reduce data skewness and potential measurement variance. Additionally, measuring biomarkers at only two time points may not fully capture dynamic inflammatory changes, warranting consideration in future research design and interpretation.

A key methodological consideration in this study was the use of an empty canal in the LLLT group compared to the Calcium Hydroxide group. While this created distinct biological environments for sampling, this design was essential to isolate the specific bio-stimulatory effect of the laser and determine its potential as a standalone alternative for symptoms management. It is also important to note the inherent difference in mechanisms between these two interventions. Although Calcium Hydroxide group exhibited a greater numerical reduction in Substance P, the lack of statistical significance suggests clinical comparability for acute pain and inflammatory modulation. However, these results should be interpreted with caution; CH remains the gold standard for its well-established antimicrobial properties, a factor not evaluated in the present study. Consequently, while LLLT appears to be a viable single-visit option for managing the symptomatic and biochemical aspects of apical periodontitis, it may not serve as a direct substitute for the long-term disinfecting role of traditional intracanal medicaments.

Future studies are recommended to include multirooted teeth to improve generalizability and to adopt enhanced blinding protocols, such as incorporating placebo laser application in the Calcium Hydroxide group, to minimize performance bias. Increasing sample size and incorporating additional time points for biomarker analysis may provide a clearer understanding of inflammatory dynamics. Additionally, integration of psychosocial and objective pain assessments is recommended. While our study isolated the effects of LLLT by using an empty canal, future research should investigate the synergistic potential of combining LLLT with various intracanal medicaments. Such studies could determine if the bio-stimulatory effects of laser therapy can enhance the antimicrobial efficacy of CH, potentially offering a superior hybrid protocol for complex endodontic cases. Finally, investigating LLLT’s effects on asymptomatic apical periodontitis may further elucidate its therapeutic potential.

## Conclusion

LLLT may be considered a viable single-visit treatment option and an effective alternative to Calcium Hydroxide for the management of Symptomatic Apical Periodontitis, as both demonstrated comparable clinical pain relief and biochemical modulation of IL-8 and Substance P. However, as this study focused on immediate symptomatic relief, further research is required to assess the efficacy of LLLT in asymptomatic lesions and its long-term impact on periapical healing outcomes.

## Supplementary Information

Supplementary Material 1.

## Data Availability

The datasets used and/or analyzed during the current study are available from the corresponding author on reasonable request.

## Abbreviations

AP: Apical periodontitis
ATP: Adenosine triphosphate
CH: Calcium Hydroxide
ELISA: Enzyme-linked immunosorbent assay
IL-8: Interleukin 8
LLLT: Low level laser therapy
NaOCl: Sodium hypochlorite
PA: Periapical
PBS: Phosphate buffered saline
POP: Post-operative pain
PS: Periapical sample
SP: Substance P
TNF: Tumor necrosis factor
VAS: Visual analogue scale

## References

[1] Sathorn C, Parashos P, Messer H. The prevalence of postoperative pain and flare-up in single- and multiple-visit endodontic treatment: a systematic review. Int Endod J. 2008;41(2):91–9. 17956561 10.1111/j.1365-2591.2007.01316.x

[2] de Oliveira Damasceno C, da Silveira Bueno CE, De Martin AS, Pelegrine RA, Villela AM, Ruivo LM, et al. Factors Associated with Post-Endodontic Treatment Pain Performed by Students in an Endodontic Graduate Program. Iran Endod J. 2020;15(4):221–6. 36704112 10.22037/iej.v15i4.26214 PMC9709830

[3] Rechenberg D-K, Bostanci N, Zehnder M, Belibasakis GN. Periapical fluid RANKL and IL-8 are differentially regulated in pulpitis and apical periodontitis. Cytokine. 2014;69(1):116–9. 25022970 10.1016/j.cyto.2014.05.014

[4] Bayliss WM. On the origin from the spinal cord of the vaso-dilator fibres of the hind-limb, and on the nature of these fibres. J Physiol. 1901;26(3–4):173–209. 16992575 10.1113/jphysiol.1901.sp000831 PMC1540518

[5] Sacerdote P, Levrini L. Peripheral mechanisms of dental pain: the role of substance P. Mediat Inflamm. 2012;2012(1):951920. 10.1155/2012/951920 PMC3306979 22474402

[6] Mester E, Ludany G, Selyei M, Szende B, Total GJ. The stimulating effect of low power laser rays on biological systems. Laser Rev. 1968;1:3. https://www.osti.gov/biblio/4836455

[7] Memon NA, Memon MR, Feroz A. Assessment of the interappointment pain by using two different intracanal medicaments. Pakistan Oral Dent J. 2013;33(1):145–50. https://www.pakistanoralanddentaljournal.com/index.php/podj

[8] Shimauchi H, Miki Y, Takayama S, Imai T, Okada H. Development of a quantitative sampling method for periapical exudates from human root canals. J Endod. 1996;22(11):612–5. 9198418 10.1016/s0099-2399(96)80032-x

[9] Arslan H, Yildiz ED, Koseoglu S. Effects of endodontic treatment on salivary levels of CGRP and substance P: a pilot study. Restor Dent Endod. 2020;45(3):e40. 32839721 10.5395/rde.2020.45.e40 PMC7431939

[10] Hopewell S, Chan AW, Collins GS, Hróbjartsson A, Moher D, Schulz KF, et al. CONSORT 2025 Statement: updated guideline for reporting randomised trials. BMJ. 2025;388:e081123. 10.1136/bmj-2024-081123. 10.1136/bmj-2024-081123 PMC11995449 40228833

[11] Tunér J, Hode L. The new laser therapy handbook: a guide for research scientists, doctors, dentists, veterinarians and other interested parties within the medical field. Grängesberg: Prima Books AB; 2010.

[12] Karu TI. Mechanisms of interaction of monochromatic visible light with cells. In: Effects of Low-Power Light on Biological Systems. SPIE Proceedings. 1996;2630:2. 10.1117/12.230023.

[13] Tadakuma T. Possible application of the laser in immunobiology. Keio J Med. 1993;42(4):180–2. 8126975 10.2302/kjm.42.180

[14] Toopalle SV, Yadav I, Gupta A, Chauhan N, Abraham D, Singh A, Sharma M. Effect of laser therapy on postoperative pain and endodontic retreatment: a systematic review and meta-analysis. Int Dent J. 2024;74(2):335–42. 37985344 10.1016/j.identj.2023.10.012 PMC10988258

[15] Kadam AS, Merwade S, Kumar Neelakantappa K, Naik SB, Brigit B, Bhumralkar SS, Naik BH. Effect of laser photobiomodulation on postoperative pain in endodontics: a systematic review. Photobiomodul Photomed Laser Surg. 2024;42(1):11– 9. 10.1089/photob.2023.0129. 10.1089/photob.2023.0129 38252493

[16] Law AS, Nixdorf DR, Aguirre AM, Reams GJ, Tortomasi AJ, Manne BD, et al. Predicting severe pain after root canal therapy in the National Dental PBRN. J Dent Res. 2015;94(3 Suppl):s37–43. 10.1177/0022034514555144 PMC4336154 25355775

[17] Alshehri AA, Alshraim RA, Abo Dawood AA. Endodontic Flare-Ups: A Study of Incidence and Related Factors. Egypt J Hosp Med. 2018;70(2):349–53.

[18] Parirokh M, Sadr S, Nakhaee N, Abbott PV, Manochehrifar H. Comparison between prescription of regular or on-demand ibuprofen on postoperative pain after single-visit root canal treatment of teeth with irreversible pulpitis. J Endod. 2014;40(2):151–4. 24461395 10.1016/j.joen.2013.09.024

[19] Ansar W, Ghosh S. Inflammation and inflammatory diseases, markers, and mediators: Role of CRP in some inflammatory diseases. In: Biology of C Reactive Protein in Health and Disease. New Delhi: Springer India; 2016. p. 67–107. 10.1007/978-81-322-2680-2_4.

[20] Chow R, Armati P, Laakso EL, Bjordal JM, Baxter GD. Inhibitory effects of laser irradiation on peripheral mammalian nerves and relevance to analgesic effects: a systematic review. Photomed Laser Surg. 2011;29(6):365–81. 21456946 10.1089/pho.2010.2928

[21] Mankar N, Burde K, Agrawal P, Chandak M, Ikhar A, Patel A. Application of Low-Level Laser Therapy in Endodontics: A Narrative Review. Cureus. 2023;15(10):e48010. 38046501 10.7759/cureus.48010 PMC10689117

[22] Fazlyab M, Esmaeili Shahmirzadi S, Esnaashari E, Azizi A, Moshari AA. Effect of low-level laser therapy on postoperative pain after single-visit root canal retreatment of mandibular molars: A randomized controlled clinical trial. Int Endod J. 2021;54(11):2006–15. 34383325 10.1111/iej.13608

[23] Ismail HH, Obeid M, Hassanien E. Efficiency of diode laser in control of post-endodontic pain: a randomized controlled trial. Clin Oral Investig. 2023;27(6):2797–804. 36662285 10.1007/s00784-023-04864-z PMC10264274

[24] Toopalle SV, Yadav I, Gupta A, Chauhan N, Abraham D, Singh A, et al. Effect of Laser Therapy on Postoperative Pain and Endodontic Retreatment: A Systematic Review and Meta-Analysis. Int Dent J. 2024;74(2):335–42. 10.1016/j.identj.2023.10.012. 10.1016/j.identj.2023.10.012 PMC10988258 37985344

[25] Asnaashari M, Ashraf H, Daghayeghi AH, Mojahedi SM, Azari-Marhabi S. Management of Post Endodontic Retreatment Pain With Low Level Laser Therapy. J Lasers Med Sci. 2017;8(3):128–31. 29123632 10.15171/jlms.2017.23 PMC5662501

[26] Caviedes-Bucheli J, Azuero-Holguin MM, Correa-Ortiz JA, Aguilar-Mora MV, Pedroza-Flores JD, Ulate E, et al. Effect of experimentally induced occlusal trauma on substance P expression in human dental pulp and periodontal ligament. J Endod. 2011;37(5):627–30. 21496661 10.1016/j.joen.2011.02.013

[27] Huang Y-Y, Sharma SK, Carroll J, Hamblin MR. Biphasic dose response in low level light therapy–an update. Dose-response. 2011;9(4):dose–response. 11 – 009. Hamblin. 10.2203/dose-response.11-009.Hamblin PMC3315174 22461763

[28] Doganay Yildiz E, Arslan H. Effect of Low-level Laser Therapy on Postoperative Pain in Molars with Symptomatic Apical Periodontitis: A Randomized Placebo-controlled Clinical Trial. J Endod. 2018;44(11):1610–5. 30144985 10.1016/j.joen.2018.07.002

[29] Rao RD, Shivangi S, Jain AK, Verma MR, Guha A, Langade D. Comparative evaluation of postoperative pain following chemomechanical preparation of single-rooted nonvital teeth with symptomatic apical periodontitis with and without laser irradiation: A double-blind randomized placebo controlled clinical trial. J Conserv Dent. 2022;25(6):610–5. 36591592 10.4103/jcd.jcd_276_22 PMC9795699

[30] Sağlam H, Aladağ H. Comparison of ıntracanal ozone and low-level laser therapy on postoperative pain in vital teeth with symptomatic apical periodontitis:placebo-controlled randomize trial. Lasers Med Sci. 2023;38(1):227. 37776342 10.1007/s10103-023-03881-4

[31] Rai V, Moellmer R, Agrawal DK. The role of CXCL8 in chronic nonhealing diabetic foot ulcers and phenotypic changes in fibroblasts: a molecular perspective. Mol Biol Rep. 2022;49(2):1565–72. 35044539 10.1007/s11033-022-07144-3

[32] Arslan H, Doğanay E, Karataş E, Ünlü MA, Ahmed HMA. Effect of Low-level Laser Therapy on Postoperative Pain after Root Canal Retreatment: A Preliminary Placebo-controlled, Triple-blind, Randomized Clinical Trial. J Endod. 2017;43(11):1765–9. 28967495 10.1016/j.joen.2017.06.028

[33] Basso FG, Pansani TN, Soares DG, Scheffel DL, Bagnato VS, de Souza Costa CA, et al. Biomodulation of Inflammatory Cytokines Related to Oral Mucositis by Low-Level Laser Therapy. Photochem Photobiol. 2015;91(4):952–6. 25735212 10.1111/php.12445

[34] Zwiri AMA, Ahmad WMAW, Asif JA, Phaik KS, Husein A, Kassim NK, et al. A Randomized Controlled Trial Evaluating the Levels of the Biomarkers hs-CRP, IL-6, and IL-8 in Patients with Temporomandibular Disorder Treated with LLLT, Traditional Conservative Treatment, and a Combination of Both. Int J Environ Res Public Health. 2022;19(15):8987. 10.3390/ijerph19158987. 10.3390/ijerph19158987 PMC9332699 35897358

[35] Doganay Yildiz E, Arslan H, Koseoglu S, Arabaci T, Yildiz DA, Savran L. The effect of photobiomodulation on total amount of substance P in gingival crevicular fluid: placebo-controlled randomized clinical trial. Lasers Med Sci. 2019;34(3):517–23. 30171442 10.1007/s10103-018-2625-3

